# Income gap between male and female psychiatric nurses in China: A national survey

**DOI:** 10.1111/inr.12996

**Published:** 2024-06-26

**Authors:** Jingyang Gu, Yang Cheng, Mengyue Gu, Song Wang, Yudong Shi, Lei Xia, Feng Jiang, Huanzhong Liu, Yilang Tang

**Affiliations:** ^1^ Department of Psychiatry Chaohu Hospital of Anhui Medical University Hefei China; ^2^ Department of Psychiatry School of Mental Health and Psychological Sciences Anhui Medical University Hefei China; ^3^ School of International and Public Affairs Shanghai Jiao Tong University Shanghai China; ^4^ Institute of Healthy Yangtze River Delta Shanghai Jiao Tong University Shanghai China; ^5^ Department of Psychiatry and Behavioral Sciences Emory University Atlanta USA; ^6^ Atlanta VA Medical Center Decatur Georgia USA

**Keywords:** Gender income gap, income, job satisfaction, night shifts, psychiatric nurses

## Abstract

**Aim:**

To investigate gender differences in the actual and expected income among psychiatric nurses in China.

**Background:**

Although studies have shown that male nurses earn more than female nurses in other countries, there are no published data regarding gender income differences among psychiatric nurses in China.

**Methods:**

We conducted a cross‐sectional study involving 41 representative psychiatric hospitals in China. Demographic, income, and job‐related data were analyzed using the inverse probability of treatment weighting (IPTW) based on the propensity score.

**Findings:**

The sample included 9256 psychiatric nurses, and nearly four‐fifths (79.3%) were female. Males earned slightly higher average monthly incomes than female nurses, while initial analysis showed no significant overall gender income difference (*p *> 0.05). Notably, most participants (92.5%) desired an income increase of at least 10%, with over half (56.2%) expressing significant dissatisfaction with their current income. After adjustment using propensity score combined with IPTW, females in the junior and mid‐level groups had significantly lower income than their male counterparts (all *p* < 0.01), despite having different night shift patterns. However, there were no significant gender differences in actual or expected income among senior‐level psychiatric nurses (*p* > 0.05).

**Conclusion:**

A majority of psychiatric nurses in China express dissatisfaction with their current incomes and expect higher incomes. Male nurses earned significantly more than female nurses in the junior and mid‐level professional groups, potentially due to their differences in night shifts.

**Implications for nursing policy and health policy:**

Policymakers and hospital administrators should optimize the income structures of nurses and develop targeted policies to address the gender income gap. Improving nurse income has the potential to enhance motivation and satisfaction within the profession.

## INTRODUCTION

The gender income gap is a persistent and widespread phenomenon affecting various sectors and occupations (Litman et al., 2020; Resnick, 2020). It refers to the differences in income between men and women for equivalent work or workload (Ganguli et al., 2022). Despite being a traditionally female‐dominated profession, nursing exhibits a notable gender income gap. In nursing jobs, men continue to earn more than women, regardless of their education, age, certification, and experience levels. A survey conducted on 6591 nurses working at least 35 hours per week revealed that male registered nurses in the United States earn approximately $7000—12,000 more per year than their female counterparts (Greene et al., 2017). Another survey of a national sample of registered nurses (*n* = 87,903) by Muench et al. (2015) also showed that the gender income gap varied by work setting, clinical specialty, and job position. The gender income gap in the United Kingdom has not significantly changed over time. Muench et al. (2016) found evidence of motivational differences in career aspirations between female and male nurses when exploring possible explanatory factors for this gap. Similarly, among practicing nurses in Peru, male nurses earned 9% more per month than female nurses after controlling for region, years of service, specialty, type of institution, average hours worked per week, type of contract, and the number of children raised (Rosas et al., 2019). In an analysis fully adjusted for demographic variables, human capital, and geographical characteristics, male nurses in the German labor market earned about 9.3% more than female nurses, but subsequent analyses found no support for gender differences (Muench & Dietrich, 2019). In China, there is a lack of published data on the gender income gap among nurses. A recent national survey conducted by Zhou et al. (2019) examined the income of nurses in public hospitals in China. The study found that nurses in tertiary public hospitals reported an actual annual income (after‐tax) of 100,000 RMB (US$14496), which was significantly lower than their expected income. Additionally, only 43.6% of participants reported being satisfied with their income. However, the study did not address gender disparity between female and male nurses. Of note, the gender income gap in nursing appears to have persisted both during and post–COVID‐19 pandemic in many countries, despite significant efforts to address it. Female nurses experienced a high rate of turnover compared to males during the pandemic. To address this issue, it has been suggested that healthcare institutions should implement a policy of equal income for both genders (Dill & Frogner, 2024). Following a public debate in the Finnish news media, the COVID‐19 crisis brought attention to the economic and political requirements and conditions of care work. This has led to a deeper and more diverse discussion on the wages of nursing professionals and the gender pay gap. However, achieving wage equality remains a challenge (Kinnunen, 2024).

It is established that nurses working in different settings and departments earn differently. Those working in higher acuity specialties (such as emergency departments, intensive care units, surgical units, etc.) often receive higher income (Li et al., 2018). However, limited research has examined the issue of income gaps within a specific department like psychiatry. Generally, there are more female nurses than male nurses in healthcare. However, in certain occupations, such as mental health, the proportion of male nurses is higher than in others (Hanrahan & Aiken, 2008). Income disparity contributes to dissatisfaction and high turnover among nurses (Pishgooie et al., 2019; Xu et al., 2020). Hence, it is imperative to investigate whether a gender disparity exists in the income of Chinese psychiatric nurses and to identify potential interventions to address this issue.

This study draws upon research by Han et al. (2022), who examined gender income gaps among psychiatrists in China. Their study, which used inverse probability of treatment weighting (IPTW) based on propensity scores to control for potential bias, found that male psychiatrists earned significantly more than their female counterparts. IPTW enables post‐hoc randomization by adjusting for covariates such as work environment and demographics to control for bias (Allan et al., 2020; Lei et al., 2020). However, this statistical method is rarely used to justify gender disparities in nurses' income. The study collected data from a national survey of psychiatric nurses working in 41 tertiary psychiatric hospitals in China. Propensity score‐based IPTW was used to control for potential bias caused by fundamental demographic covariates, such as age, years of experience, marital status, and education level. Our primary aim is to examine gender differences in both actual and expected income among psychiatric nurses in China. The findings will offer valuable insights for healthcare professionals regarding income disparities and potentially inform the development of more sensible and equitable income policies.

## METHODS

### Design and participants

We used a locally developed questionnaire to collect the following data, categorized into three categories: (1) Demographic data: age, sex, work years, marital status (married/cohabitation, unmarried/divorced), children (yes or no), and education level (college, bachelor's degree, master's degree or above); (2) Income data: actual monthly income after taxes, their income expectations (whether they expected a decrease of at least 10%, no change, or an increase of at least 10%), and their income dissatisfaction (whether low income was the main reason for being dissatisfied with their current job or not). (3) Job‐related data: whether they held an administrative position (yes or no), professional levels (ranks) (junior, mid‐level, senior), working days per week, working hours per day, and night shifts per month). The professional levels of nurses in China consist of three categories: junior (a newly registered nurse), middle (a supervisor with managerial and supervisory team leadership responsibilities), and senior (hospital‐ or system‐level leadership responsibilities, or responsible for mentoring nurses, teaching or research). Typically, senior nurses have longer tenures and often hold managerial positions (Lu et al., 2021).

Before conducting the survey, a workshop on questionnaire design was held on October 16, 2020, involving project team members and experts. A pilot test and a pre‐survey (November 27 to December 1, 2020) with 20 healthcare professionals informed revisions. This pre‐testing assessed question clarity, option presentation, and participant feedback, leading to further refinements and additional instructions.

The survey was conducted during the COVID‐19 pandemic (January–March 2021) via WeChat, a popular messaging app in China. The questionnaire was distributed to psychiatric nurses through the “Healthy China” of the National Health Commission of China (NHC). It involved 41 psychiatric hospitals across 29 provinces in mainland China, representing the national population of psychiatric nurses working in top‐tier psychiatric hospitals. Each participant's WeChat account could only submit the survey once to avoid data duplication. Informed consent was obtained from each participant, and their anonymity and privacy were ensured. Participants were presented with the informed consent form before beginning the survey, and their completion of the survey implied consent.

### Statistical analysis

Data analyses were performed using the Statistical Package for Social Sciences (SPSS, version 23) and Rstudio (2022.07.2 + 576). Descriptive statistics were calculated to summarize hospital information, income, and sample distribution. To investigate the association between gender and income, a multivariate linear regression model was constructed. This model used self‐reported mean monthly income as the outcome variable (dependent variable), gender as the primary explanatory variable, and hospital location as a fixed effect to account for potential location‐based variations. To mitigate the inherent limitations of observational data and approximate randomization, we applied the IPTW based on the propensity score. The IPTW assigns a weight of 1/(propensity score) to a female, and a weight of 1/(1‐propensity score) to a male, using the standardized mean difference (SMD) as a criterion. We considered pruning to be optimal when SMD < 0.1 (Tian et al., 2024). After adjusting for confounding factors such as demographic data, we balanced the general data of female and male nurses and conducted a regression analysis to examine the gender differences in the income of different professional ranks. As the professional title and expected income of psychiatric nurses were rank‐ordered variables, the Kendall correlation was used to test whether there was a difference in the expected income of nurses with different professional ranks.

### Ethical approval

The Ethics Committee of Chaohu Hospital, Anhui Medical University reviewed and approved the study (approval number 202002‐kyxm‐02).

## RESULTS

### Sample characteristics

A total of 9335 participants were approached for this survey across different regions in China. After eliminating data with obvious logical errors and excluding abnormal data outside the 1st and 99th quartiles (*n* = 79) (Han et al., 2022), the final analysis included data from 9256 participants. Nearly four‐fifths (79.6%) were female. The participants were divided into three professional categories: junior (5426, 58.6%), mid‐level (3208, 34.7%), and senior (622, 7.3%). Notably, junior and mid‐level nurses were predominantly female, with most holding a college/bachelor's degree, being unmarried, and working fewer night shifts compared with their male counterparts. Table 1 presented the distribution of gender, education level, marital status, and night shift frequency across the professional categories.

**TABLE 1:** Demographic data of 9256 psychiatric nurses in 41 representative psychiatric hospitals in China.

	No. (%)			Weighted			
Participants	Female	Male	*p* values^a^	Female	Male	SMD	*p* values^b^
Overall	7365 (79.57)	1891 (20.43)	NA	9277	9088		NA
Age (mean, SD)	35.85 (8.67)	33..79 (8.35)	<0.001	35.42 (8.57)	35.19 (8.81)	0.027	0.426
Working years (mean, SD)	14.11 (9.75)	11.55 (9.29)	<0.001	13.56 (9.62)	13.14 (9.83)	0.043	0.210
Working days per week (mean, SD)	5.05 (0.65)	5.01 (0.67)	0.006	5.04 (0.66)	5.04 (0.65)	0.004	0.874
Working hours per day (mean, SD)	8.29 (1.27)	8.38 (1.41)	0.006	8.31 (1.30)	8.30 (1.35)	0.004	0.885
Night shifts per month (mean, SD)	3.96 (3.93)	5.87 (3.73)	<0.001	4.39 (4.16)	4.54 (3.71)	0.037	0.216
Marital status, *n* (%)							
Unmarried/divorce	1827 (24.81)	596 (31.52)	<0.001	2432.13 (26.22)	2417.81 (26.60)	0.009	0.749
Married/cohabitated	5538 (75.19)	1295 (68.48)	<0.001	6844.67 (73.78)	6670.03 (73.40)		
Children, *n* (%)							
Yes	5255 (71.35)	1170 (61.87)	<0.001	6433.64 (69.35)	6205.64 (68.29)	0.023	0.409
No	2110 (28.65)	721 (38.13)		2843.16 (30.65)	2882.19 (31.71)		
Administrative position (%)							
Yes	494 (6.71)	108 (5.71)	0.130	602.82 (6.50)	595.01 (6.55)	0.002	0.951
No	6871 (93.29)	1783(94.29)		8673.97(93.50)	8492.82 (93.45)		
Education, *n* (%)							
College/	1960 (26.61)	735 (38.87)	<0.001	2704.00 (29.15)	2619.45 (28.82)	0.023	0.960
Bachelor's degree	5328 (72.34)	1140 (60.29)		6478.80 (69.84)	6372.02 (70.12)		
Master's degree or above	77 (1.05)	16 (0.84)		93.99 (1.01)	96.36 (1.06)		
Professional rank, *n* (%)							
Junior	4025 (54.65)	1401 (74.09)	<0.001	5442.24 (58.67)	5332.60 (58.68)	0.021	0.835
Middle	2744 (37.26)	464 (24.54)		3212.69 (34.63)	3191.28 (35.12)		
Senior	596 (8.09)	26 (1.37)		621.87 (6.70)	563.95 (6.21)		

*Note*: NA, not applicable; SMD, standardized mean difference. Generally, when SMD < 0.1, the trimming effect is considered to be better.

^a^ *p* values were obtained from Pearson chi‐squared tests comparing percentages or from two‐tailed *t*‐tests comparing means between females and males.

^b^ *p* values were obtained from a regression that compared data between females and males after using the inverse probability of treatment weighting (IPTW) based on the propensity score.

### Income differences by gender and professional rank

The study found that the average monthly after‐tax income for male nurses (8855 RMB; $1373) was slightly higher than that for females (8771 RMB; $1359). Although initial analysis showed no significant overall gender income difference (*p *> 0.05), a closer look showed a significant gender income gap in junior and mid‐level nurses, but not in senior ones. However, after adjusting for potential confounding factors using propensity scores, the gender income gap became statistically significant (*p *< 0.001) for the entire sample. The adjusted analysis confirmed the income disparity for junior and mid‐level nurses (*p *< 0.001), but not for senior nurses (*p *> 0.05) (refer to Table 2). To investigate potential factors associated with the gender income gap, we conducted an adjusted data analysis that revealed significant gender differences in night shifts among nurses in various professional ranks (*p *< 0.05) (see Table 2).

**TABLE 2:** Income levels and night shifts by gender and professional ranks in 9256 psychiatric nurses.

Monthly income	Mean (SD)		[F–M] (95% CI)		[F–M] (95% CI)	
	Female	Male	Unweighted	*p* values^a^	Weighted	*p* values^b^
Overall	8749.04 (4436.99)	8854.73 (4454.07)	−105 (−331 to 119)	0.356	871 (654–1088)	<0.001
Junior	7670.54 (3947.10)	8101.81 (4093.10	−431 (−678 to −185)	<0.001	774 (562–986)	<0.001
Middle	9900.19 (4565.52)	10,932.83 (4641.30)	−1033 (−1489 to −576	<0.001	971 (408–1533)	<0.001
Senior	10,732.66 (4926.98)	12,339.74 (6169.01)	−1607 (−4126 to 912)	0.108	2192 (−8038 to 12,422)	0.675
Night shifts						
Overall	3.96 (3.93)	5.87 (3.73)	−1.91 (−2.11 to −1.72)	<0.001	2.46 (2.19–2.73)	<0.001
Junior	5.00 (3.90)	6.43 (3.48)	−1.43 (−1.64 to −1.21)	<0.001	2.36 (2.06–2.65)	<0.001
Middle	3.06 (3.71)	4.43 (3.95)	−1.37 (−1.76 to −0.99)	<0.001	2.74 (2.09–3.39)	<0.001
Senior	1.04 (2.10)	1.69 (3.04)	−0.65 (−1.89 to 0.58)	0.129	7.31 (0.48–14.15)	0.036

^a^ *p* values were obtained from two‐tailed *t*‐tests comparing means between females and males.

^b^ *p* values were obtained from a regression that was adjusted for the IPTW weights based on the propensity score.

### Expected income and professional rank

Kendall's correlation analysis (Hicks et al., 2023) was used to assess the expected income data in different professional ranks. The results showed a significant gender difference but with a very low Kendall correlation coefficient (tau = 0.032). Additionally, a separate analysis by gender yielded similarly low Kendall coefficients (tau = 0.031 for females and tau = 0.043 for males, respectively), indicating a weak, if any, correlation between expected income and professional ranks (see Table 3).

**TABLE 3:** The gap between expected income and professional ranks.

	Professional rank			Expect income			Kendall tau^a^	*p* values^b^
	Junior	Middle	Senior	Down > 10%	Unchanged	Up >10%		
Overall	5426	3208	622	171	377	8808	0.032	0.001
Females	4025	2744	596	132	229	7004	0.031	0.005
Males	1401	464	26	39	48	1804	0.043	0.058

^a^ The Kendall coefficients with Kendall correlation analysis, the closer the tau value is to 1, the stronger the correlation.

^b^ The result of the Kendall correlation analysis according to the professional ranks.

### Nurse satisfaction with income

Low income was the most commonly reported cause of dissatisfaction among nurses, with over half (56.2%) expressing this concern. Furthermore, the majority (92.5%) desired to receive an increase in income by at least 10%.

## DISCUSSION

This study is the first national survey investigating the gender income gap among psychiatric nurses in China. The data used in this study were obtained from the most recent national survey, and the participants were from top‐tier psychiatric hospitals. Although the initial analysis found no significant overall gender income differences in the entire sample, a closer look identified a disparity among junior and mid‐level nurses but not among senior nurses. The adjusted regression analysis using propensity scores‐based IPTW further confirmed this pattern. These findings suggest that the gender income gap may be more pronounced in early career stages.

Previous studies suggested that multiple factors may contribute to the gender income gap. These include individual factors such as age, education, professional rank, and seniority (Dousin et al., 2021); work‐related factors such as work hours per week, work intensity, and the nature of the provider‐patient relationship (Rosas et al., 2019); and hospital factors such as the type and size of hospitals, and proportion of personnel expenditure (Lu et al., 2021). The gender income gap is often influenced by the occupational choices made by both females and males. According to Schneider et al. (2022), the gender income gap tends to be smaller in occupations with a higher ratio of male employees. Sociocultural and structural factors may explain the gender income gap, which can limit female's income in terms of education level and across most sectors. Although higher education can increase individual income, it does not effectively address the gender income gap. Research shows that females tend to earn less than males in jobs that are traditionally associated with males, even when they have the same level of qualifications (Gauci et al., 2023; Hoff & Lee, 2021). However, it is important to note that higher income for male nurses may be influenced by different motivations for career aspirations. Research has shown that males are more likely to change jobs for higher incomes than females (Muench et al., 2016; Wilson et al., 2016). It is expected that income scales would compensate for the effects of external selection and gender discrimination in income for male nurses (Muench & Dietrich, 2019). It is crucial to maintain income equity and avoid gender discrimination in the nursing profession. Other social and cultural factors may also contribute to this difference. For example, male nurses may experience greater financial burdens early in their careers due to societal expectations around marriage, homeownership, child‐rearing, and career progression.

It is important to provide some background information about the participants and the income structure in China. The study participants were all public hospital employees in China, and their incomes were primarily based on their education, certificates, and professional titles. The compensation for healthcare employees typically comprises three components: base income, monthly bonus income (which can often exceed the base income and is based on workload and additional responsibilities), and other income (such as income for teaching and research) (Zhang & Liu, 2018). Any gender income gap is likely due to factors other than structured base income, such as a hidden gender gap in bonus distributions, of which the night shift bonus is a significant component. To investigate factors contributing to the gender income gap in this study, we monitored the night shifts of nurses at various professional levels. We found that junior and mid‐level nurses worked more night shifts than senior nurses. Additionally, a significantly higher number of male nurses worked more night shifts than their female counterparts. It is possible that the additional bonus income received for working more night shifts may have contributed to the gender income gap. Male nurses are the primary workforce for psychiatric night shifts in psychiatric hospitals, as reported previously (Yang et al., 2018). According to Kodal et al. (2018), male nurses have a higher acceptance of psychiatric work than female nurses, which may result in higher compensation. Therefore, male nurses may be compensated more appropriately.

In this study, a majority (56.2%) of participants identified low income as the cause of their dissatisfaction. Additionally, the vast majority of participants (92.5%) expressed a desire for higher income. No significant correlations were found between expected income and professional rank. Previous studies have indicated that income is a critical factor affecting job satisfaction (Hu et al., 2020; Seo et al., 2016). Wubetie et al. (2020) found that income inequality can result in low morale, reduced work enthusiasm, and increased turnover intention. Psychiatric nurses play a crucial role in preventing and treating mental illness as critical participants in the mental healthcare system. However, the gender income gap negatively affects their job satisfaction and professional identity (Wang et al., 2022). Notably, China faces a critical shortage of psychiatric nurses, with only an average of 3.77 psychiatric nurses per 100,000 population, significantly lower than in middle‐ and high‐income countries (Marć et al., 2019). With China's rapidly aging population, the demand for psychiatric care for the elderly is increasing (Zhang et al., 2022). Therefore, it is crucial to address the income gap for nurses to stabilize nursing staff.

This study found that the number of night shifts worked by nurses is one of the factors contributing to the gender income gap, along with other possible factors. It is important to note that an in‐depth study of nurses' income structure was not conducted, which may also impact their income levels. This will be explored further in future research.

## LIMITATIONS

This first national study had a large number of participants and comprehensive data collection on demographics and work‐related factors, coupled with rigorous statistical methods to control for potential confounding factors. However, a few limitations should be acknowledged. Firstly, the income data relied on self‐reporting, potentially introducing reporting bias. Secondly, the sample was exclusively drawn from psychiatric nurses in tertiary psychiatric hospitals, thereby limiting the generability of findings to psychiatric hospitals in smaller cities and rural areas. Thirdly, despite meticulous efforts to control for as many factors as possible, we did not gather information on the participants' exact work settings within the hospital, such as the emergency department, outpatient or inpatient units, closed or open units, and so on.

### Implications for nursing and health policy

Policymakers and hospital administrators should be aware of the income gap between male and female nurses and take steps to address it. Our study found that differences in the number of night shifts worked were one factor influencing the gender income gap, but there may be other contributing factors as well. To promote fairness and address income disparity, hospital administrators should optimize and restructure the income structure of the nursing workforce. Face‐to‐face interviews should be conducted to determine the actual income of nurses and to identify potential reasons for paying male nurses more than female nurses. To address the internal imbalance caused by income disparity, it is important to ensure that all nurses are fairly compensated and have equal opportunities for income and promotion. This will enhance their motivation and job satisfaction, decrease the nursing staff turnover rate, and maintain the stability of the nursing workforce.

## CONCLUSION

This study presents the first national survey of income levels and the gender income gap among psychiatric nurses in China. The majority of surveyed psychiatric nurses surveyed expressed dissatisfaction with their current income and desired an increase in income. In China, the overall income level between male and female nurses was found to be comparable. However, it was discovered that female psychiatric nurses in the junior and middle professional ranks earn significantly less than their male peers after controlling for confounding factors. One possible explanation for this gender income gap is the difference in night shift schedules between genders. Policymakers and hospital administrators need to be aware of the issue and take action to address it.

## AUTHOR CONTRIBUTIONS

*Study design*: Huanzhong Liu and Feng Jiang. *Data collection*: Jingyang Gu, Yang Cheng, Mengyue Gu, Song Wang, Yudong Shi, and Lei Xia. *Data analysis*: Jingyang Gu. *Study supervision*: Huanzhong Liu. *Manuscript writing*: Jingyang Gu and Yang Cheng. *Critical revisions for important intellectual content*: Huanzhong Liu, Feng Jiang, and Yilang Tang.

## CONFLICT OF INTEREST STATEMENT

The authors declared that there are no conflicts of interest about the research and authorship.

## ETHICAL STATEMENT

The Ethics Committee of Chaohu Hospital, Anhui Medical University reviewed and approved the study (approval number 202002‐kyxm‐02).

## Data Availability

The data results of this study have been presented in this manuscript. The original data involved in this study can be obtained by contacting the corresponding author.
